# NiFe_2_O_4_ Material on Carbon Paper as an Electrocatalyst for Alkaline Water Electrolysis Module

**DOI:** 10.3390/mi15010062

**Published:** 2023-12-28

**Authors:** Ying-Chyi Wang, Shuo-En Yu, Yu-Lun Su, I-Chun Cheng, Yi-Cheng Chuang, Yong-Song Chen, Jian-Zhang Chen

**Affiliations:** 1 Institute of Applied Mechanics, National Taiwan University, Taipei City 106319, Taiwan; r10543002@ntu.edu.tw (Y.-C.W.); r11543032@ntu.edu.tw (Y.-L.S.); 2 Graduate School of Advanced Technology, National Taiwan University, Taipei City 106319, Taiwan; f11k45004@ntu.edu.tw; 3 Department of Electrical Engineering, Graduate Institute of Photonics and Optoelectronics, National Taiwan University, Taipei City 106319, Taiwan; iccheng@ntu.edu.tw; 4 Innovative Photonics Advanced Research Center (i-PARC), National Taiwan University, Taipei City 106319, Taiwan; 5 Department of Mechanical Engineering, Advanced Institute of Manufacturing with High-Tech Innovations, National Chung Cheng University, Chiayi County 621301, Taiwan; yicheng@ccu.edu.tw (Y.-C.C.); imeysc@ccu.edu.tw (Y.-S.C.); 6 Advanced Research Center for Green Materials Science and Technology, National Taiwan University, Taipei City 106319, Taiwan

**Keywords:** alkaline water electrolysis, hydrothermal method, electrocatalyst, NiFe, Ru

## Abstract

NiFe_2_O_4_ material is grown on carbon paper (CP) with the hydrothermal method for use as electrocatalysts in an alkaline electrolyzer. NiFe_2_O_4_ material is used as the anode and cathode catalysts (named NiFe(+)/NiFe(−) hereafter). The results are compared with those obtained using CP/NiFe as the anode and CP/Ru as the cathode (named NiFe)(+)/Ru(−) hereafter). During cell operation with NiFe(+)/Ru(−), the current density reaches 500 mA/cm^2^ at a cell voltage of 1.79 V, with a specific energy consumption of 4.9 kWh/m^3^ and an energy efficiency of 66.2%. In comparison, for NiFe(+)/NiFe(−), the current density reaches 500 mA/cm^2^ at a cell voltage of 2.23 V, with a specific energy consumption of 5.7 kWh/m^3^ and an energy efficiency of 56.6%. The Faradaic efficiency is 96–99%. With the current density fixed at 400 mA/cm^2^, after performing a test for 150 h, the cell voltage with NiFe(+)/Ru(−) increases by 0.167 V, whereas that with NiFe(+)/NiFe(−) decreases by only 0.010 V. Good, long-term stability is demonstrated.

## 1. Introduction

High fossil fuel consumption has resulted in a rapid increase in the carbon dioxide concentration in the air, causing serious hazards such as global warming, climate change, and air pollution [1,2,3]. To mitigate these problems, the development of green energy has become an important goal [4]. Hydrogen energy is a clean fuel. It can be used in combination with solar energy, wind energy, hydropower, and other renewable energy sources to convert excess electricity into hydrogen [5,6,7,8,9]. Further, it involves a reversible reaction that converts hydrogen into electricity [10,11,12]. The redox reactions do not emit harmful gases, making hydrogen a future energy source with great potential [13,14,15,16,17,18,19].

In hydrogen energy technology, fuel cells are important power generation devices. Fuel cells are the result of the reverse reaction of water electrolysis, using hydrogen and oxygen to produce electricity and water. Fuel cells do not produce harmful gases during the power generation process and are very friendly to the environment. This power generation method is much cleaner than those using fossil fuels. The power generation efficiency of fuel cells can reach 60%. If the waste heat generated via power generation can be recovered, the efficiency can even exceed 80%. The hydrogen, generated via water electrolysis with green energy, supplied to fuel cells can reduce carbon dioxide emissions [20,21].

An alkaline water electrolysis system is used for electrolytic hydrogen production. In this system, the oxygen evolution reaction (OER) and hydrogen evolution reaction (HER) occur at the anode and cathode, respectively [22,23,24,25,26]. In alkaline water electrolysis, the reactions that occur at the cathode and anode are as follows [27]:

Cathode: 2 H_2_O + 2 e^−^ → H_2_ + 2 OH^−^

Anode: 4 OH^−^ → 4 e^−^ + 2 H_2_O + O_2_

$$\mathrm{Overall}\text{: }H_{2}O\text{ → }H_{2}\;+\;\frac{1}{2}O_{2}$$

Compared to HER, OER involves more complex reaction pathways and is generally considered a process with higher thermodynamic and kinetic requirements in water electrolysis [28].

Precious metals such as Ir, Pt, and Ru are excellent high-efficiency electrocatalysts [29,30,31,32,33,34]; however, they are expensive and unsuitable for large-scale modules. In addition, electricity consumption usually accounts for more than 50% of the cost of the water electrolysis system [35,36]; therefore, the electrocatalysts must minimize the overpotential of the electrolysis reaction to reduce costs [37,38,39]. In this light, the development of high-performance and low-cost catalytic materials has become an important goal [40,41,42,43]. Ni is an abundant 3D transition metal on earth and has the characteristics of corrosion resistance, high stability, and high electrocatalytic activity [44]. At the same time, Fe is also an abundant transition metal in the earth’s crust. Its toxicity and cost are lower than those of cobalt and nickel, and it has rich redox properties and excellent electrocatalytic properties [45]. Therefore, Ni- and Fe-based materials were used to greatly reduce the overpotential of the OER reaction, thereby improving the performance of the overall system [46,47,48,49,50,51,52,53,54,55,56,57,58]. Moreover, many types of nanomaterials, like metal–organic frameworks (MOF) and metal oxides, have been widely investigated in recent research for alkaline water electrolysis. MOF is a material structure that has attracted much attention in recent years. In MOF, the metal central atoms are bonded by organic ligands [59,60,61]. Depending on the combination of different metals and ligands, it can form a one-dimensional, two-dimensional, or three-dimensional structure. This kind of structure features a large specific surface area, adjustable pores, and adjustable central characteristics [61]. On the other hand, due to their low cost, high abundance, and excellent corrosion resistance in alkaline environments, transition metal oxides have been extensively developed as OER (oxygen evolution reaction) catalysts [28]. Ni- and Fe-based metal oxides, like NiFe_2_O_4_, have been demonstrated as an efficient OER catalyst for anion exchange membrane water electrolysis modules [62,63]. In our synthesized material, it contains NiFe_2_O_4_ with a small proportion of NiFe-MOF.

In alkaline water electrolysis, compared to traditional powdered electrocatalysts, self-supported electrodes with catalytically active phases grown in situ on conductive substrates are more favored because they have the following advantages: Firstly, the use of a solvothermal method simplifies the electrode preparation process and reduces costs [64]. Secondly, the preparation of the electrode makes it easier to achieve surface hydrophilic/hydrophobic engineering. Hydrophilic electrodes can accelerate bubble detachment, enhance the contact between the electrocatalyst and electrolyte, and facilitate charge and ion transfer [65,66]. These advantages contribute to enhancing the catalytic activity and long-term stability of self-supported electrodes in practical high-current-density electrolysis [66]. On the other hand, carbon paper, one of the carbon materials with the advantages of porous structure, high surface area, and low cost, is currently widely used in many energy devices [28,67,68,69]. In this work, the NiFe_2_O_4_ material with a small portion of NiFe-MOF material is obtained through a hydrothermal method, which allows the oxide crystal to be directly deposited on the carbon paper substrate [70].

Among the various current hydrogen production technologies through water electrolysis, alkaline water electrolysis (AWE) for hydrogen production is still the most widely used technology for large-scale hydrogen production. AWE technology is currently relatively stable and mature, and it can use non-noble metal-based materials as catalysts. However, the operating current density is relatively low, and based on safety considerations, the pressure on both sides of the cathode and anode also needs to be controlled [27,71]. On the other hand, the proton exchange membrane water electrolysis (PEMWE) technology has high energy conversion efficiency and high purity of the output gas, and the electrolyzer can also achieve a compact stacked design [27]. The commercialization of PEM water electrolysis has encountered some significant drawbacks. For instance, PEM electrolyzers often require the use of precious metals such as Pt as the cathode catalyst and Ru/Ir-based oxides as the anode catalyst due to their corrosion resistance in acidic environments. The use of precious metal catalysts significantly increases costs and limits the widespread application of PEM water electrolysis [72]. In recent years, new concepts for alkaline water electrolysis, such as anion exchange membrane water electrolysis and alkaline zero-gap water electrolysis, have been developed to reduce the consumption of alkaline electrolyte and enhance operating current density [73,74]. With the benefit of alkaline water electrolysis and proton exchange membrane water electrolysis, the anion exchange membrane water electrolysis (AEMWE) technology has attracted lots of attention recently. The AEMWE allows the non-precious metal catalyst to operate in a relatively noncorrosive electrolyte at low temperatures and with a lower cost of equipment setup [70,75,76]. However, AEMWE is currently mostly in the experimental research and development stage. Aside from the design and fabrication of the electrocatalyst, further investigation of the power efficiency and stability is still required to improve the AEMWE system [76]. Consequently, in this work, not only has the NiFe-based electrocatalyst been studied, but also the system performance of the AEMWE system has been investigated.

In recent years, there have been many studies on the electrocatalytic layer, but most of them focus more on conducting material analysis on the electrocatalyst. Compared with electrochemical measurement, there are relatively few studies on complete electrolysis modules. Furthermore, during the manufacturing process, we try to use low-cost, low-toxic materials and simple manufacturing methods to achieve the goal of being environmentally friendly while investigating the possibility of applying the AEMWE technology on a commercial scale. In this study, we used carbon paper (CP) as a substrate for the deposition of electrocatalysts. NiFe_2_O_4_ material electrocatalysts are used in an alkaline water electrolysis system. A NiFe_2_O_4_ material is used for both the anode and cathode of the water electrolysis system (named NiFe(+)/NiFe(−) hereafter). The results are compared with those of a water electrolysis system containing NiFe as the anode and Ru as the cathode (named NiFe(+)/Ru(−) hereafter).

## 2. Experimental

### 2.1. Electrolyzer

**Figure 1:**
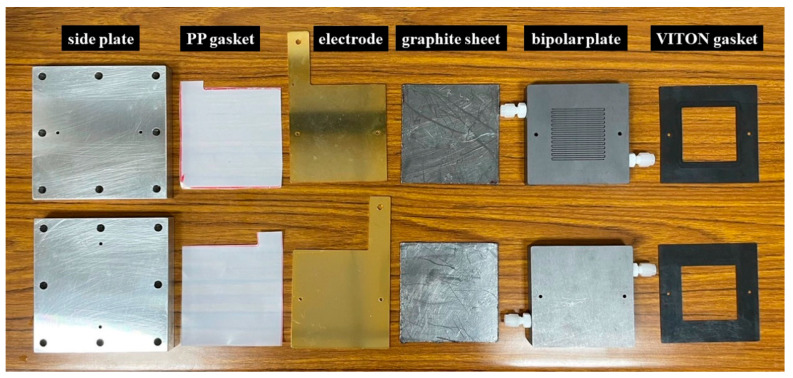
Components of electrolyzer.

Figure 1 shows the components of the electrolyzer. The system was symmetric, with the same structure on the anode and cathode sides. From the outer to the inner layer, the system consisted of an aluminum side plate, polypropylene gasket, copper electrode with gold coating, flexible graphite sheet, graphite bipolar plate, and VITON rubber gasket. Electrocatalysts on CP with a dimension of 5 cm × 5 cm were placed in the VITON rubber gaskets and covered with a 6 cm × 6 cm anion exchange membrane (Sustainion^®^ X37-50 Grade RT Membrane, Dioxide Materials, Boca Raton, FL, USA).

We tested the performance of the alkaline water electrolyzer at room temperature. The system was fed with 1 M KOH into both the anode and cathode using two peristaltic pumps at a flow rate of 10 mL/min. Before the system test, the X37-50 membrane was activated by soaking in 1 M KOH for 24 h. 

### 2.2. Preparation of NiFe Solution and Ru Solution for Hydrothermal Process

In a NiFe solution, 25 mmol of FeCl_3_·6H_2_O (purity: >99%, Sigma-Aldrich, St. Louise, MO, USA), 25 mmol of Ni(NO_3_)_2_·6H_2_O (purity: 99.999%, Sigma-Aldrich), 7 mmol of 2-aminoterephthalic acid (H_2_BDC-NH_2_) (purity: 99%, Sigma-Aldrich), 160 mL of ethanol (purity: 95%, Echo Chemical, Miaoli County, Taiwan), and 2.5 mL of acetic acid (purity: >99.5%, AUECC, Hsin-Chu City, Taiwan) were added in a Teflon autoclave reactor. The mixture solution was stirred at 200 rpm at room temperature for 30 min.

In the Ru solution, 5 mmol of RuCl_3_·3H_2_O (purity: 99.98%, Sigma-Aldrich, St. Louise, MO, USA), 80 mL of ethylene glycol (purity: 99%, Show-wa, Tokyo, Japan), and 80 mL of deionized (DI) water were added in a Teflon autoclave reactor. Then, the solution was stirred at 200 rpm at room temperature for 30 min.

### 2.3. Deposition of Electrocatalysts on CP

CP (thickness: 0.43 mm, CeTech, Taichung City, Taiwan) was cut into a 5 cm × 5 cm square as the substrate for electrocatalyst deposition. Next, it was treated in a plasma cleaner with Ar plasma at a power of 11 W, a flow rate of 5 sccm, and a chamber pressure of 0.6 torr for 1 min. Plasma treatment cleans the surface and makes CP hydrophilic.

Two pieces of CP were placed into a Teflon autoclave reactor with Ru and NiFe solutions, respectively. The autoclaves were heated in the oven at 160 °C for 16 h. After the hydrothermal processes, the samples were cleaned with DI water and dried in the oven at 80 °C.

### 2.4. Characterization of Electrocatalysts on CP

Scanning electron microscopy (SEM; JSM-IT100, JEOL, Tokyo, Japan) was used to examine the surface morphology of the electrocatalysts. An optical goniometer (model 100SB, Sindetake, Taipei City, Taiwan) was used for water contact angle measurements. A power supply (SPS-1230, GWInstek, New Taipei City, Taiwan) and a multimeter (15B, FLUKE, Everett, WA, USA) were used to measure the current and voltage of the electrolyzer. X-ray photoelectron spectroscopy (XPS; K-Alpha, Thermo Fisher Scientific, Waltham, MA, USA) with an Al-Kα X-ray source was used to analyze the chemical surface structure of the electrocatalysts. Further, X-ray diffraction (XRD; D8 Discover, Bruker, Billerica, MA, USA) was used to analyze the structure of the electrocatalysts.

## 3. Results and Discussion

### 3.1. Performance of Alkaline Water Electrolyzer

**Figure 2:**
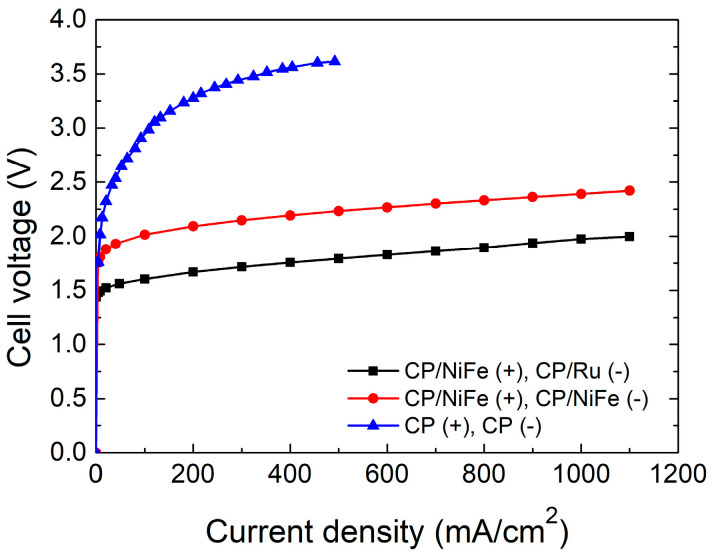
Performance cell voltage–current density curves of electrolyzers.

To test the performance of an alkaline water electrolyzer, we adjust the current value output by the power supply to observe the voltage under different current densities. During the test, the current density was adjusted upwards from zero by 100 mA/cm^2^ at each step, and each current value was held for 20 s to make sure that the voltage value was in a stable state. Figure 2 shows the performance of the electrolyzer. NiFe(+)/Ru(−) is better than NiFe(+)/NiFe(−). For NiFe(+)/Ru(−), the electrolysis reaction starts at 1.49 V, and the electrolyzer shows a current density of 500 mA/cm^2^ at a cell voltage of 1.79 V. In comparison, for NiFe(+)/NiFe(−), hydrogen and oxygen production are observed at 1.81 V, and the electrolyzer shows a current density of 500 mA/cm^2^ at a cell voltage of 2.23 V. Bare CP without electrocatalysts was tested for comparison. A comparison of the performance curves revealed that the electrocatalysts are indeed functioning. CP/Ru as a cathode electrocatalyst has better performance than that of CP/NiFe. This shows that Ru is indeed a highly efficient electrocatalyst in the HER reaction [77,78]. At the same current density, the cell voltage of the NiFe(+)/NiFe(−) system was 0.3–0.4 V higher than that of the NiFe(+)/Ru(−) system. CP/NiFe electrocatalysts can be a feasible option without the use of precious metal, and this can reduce the material cost in a practical alkaline water electrolysis system [51].

**Figure 3:**
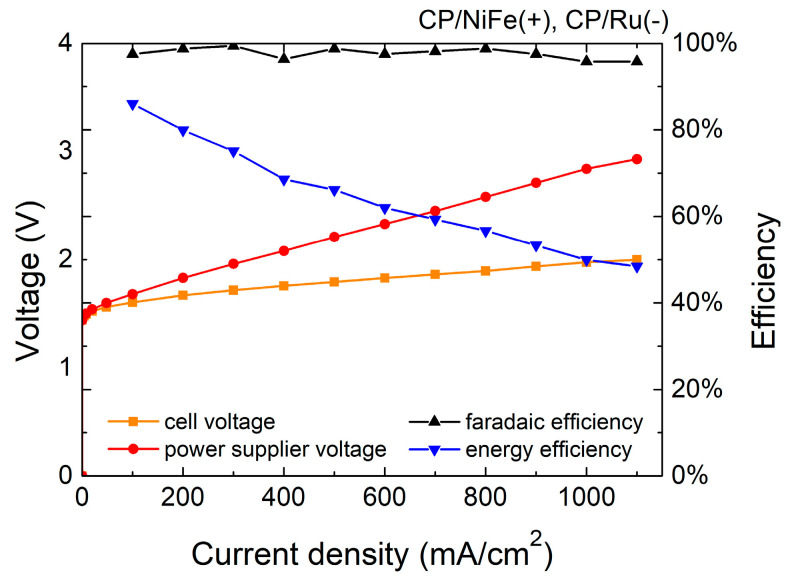
Efficiency of NiFe(+)/Ru(−) electrolyzer.

**Figure 4:**
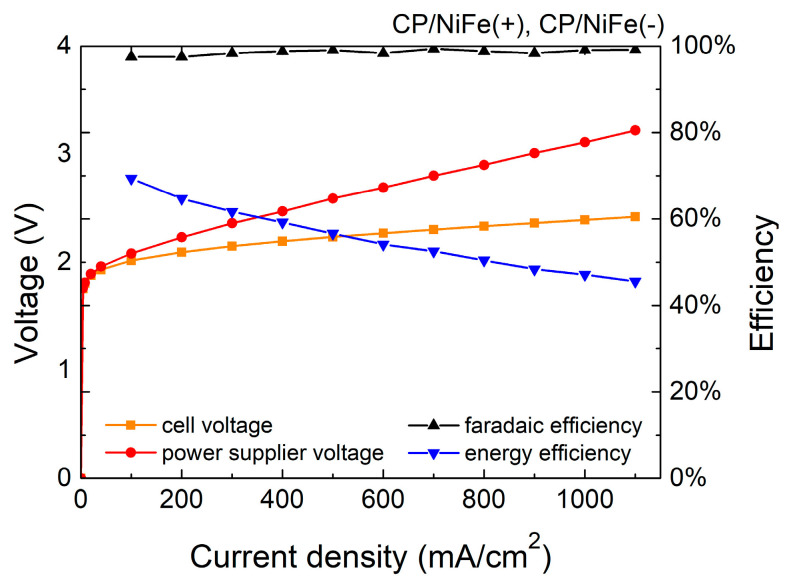
Efficiency of NiFe(+)/NiFe(−) electrolyzer.

Figure 3 and Figure 4 and Table 1 show the Faradaic efficiency (FE) and energy efficiency of the electrolyzer. FE is the ratio of the experimental hydrogen production rate to the theoretical hydrogen production rate. In this study, FE is 96–99%, indicating that most electrons were used to electrolyze water with little side reaction. The specific energy consumption of the electrolyzer of the NiFe(+)/Ru(−) system was 4.9 kWh/m^3^, and that of the NiFe(+)/NiFe(−) system was 5.7 kWh/m^3^ at 500 mA/cm^2^.

The energy efficiency (η) is calculated as follows:

$$\eta \;=\;\frac{E_{H_{2}}}{Q}\;=\;\frac{P_{H_{2}}*11.7J}{I*V_{ps}}$$

in which $E_{H_{2}}$ is the chemical energy of the produced hydrogen, *Q* is the total energy consumption of the electrolytic reaction, $P_{H_{2}}$ is the experimental hydrogen production rate, 11.7 J is the heating value of hydrogen per cm^3^ (heating value of hydrogen is 141.8 MJ/kg [79]), I is the electrolyzer current, $V_{ps}$ is the voltage of the power supply, and η of the NiFe(+)/Ru(−) system is 66.2% whereas that of the NiFe(+)/NiFe(−) system is 56.6% at 500 mA/cm^2^. In addition, the sum of the wire and contact resistance in the system was approximately 33 mohm, which inevitably caused some additional energy loss.

In electrolysis systems, an important indicator is the Faradaic efficiency. Faradaic efficiency describes the efficiency of converting electrical charge in the external circuit into the production of hydrogen/oxygen molecules through the electrolysis of water. In other words, Faradaic efficiency is the ratio of the actual gas produced to the theoretical gas produced [80]. The higher the Faradaic efficiency, the higher the proportion of electrolysis current used for the electrolysis of water. The ideal Faradaic efficiency should be 100%, which means that there are no side reactions in the system and all electrons are used for the electrolysis of water.

The Faradaic efficiency (FE) is calculated as follows:

$$\mathrm{FE}\;=\;\frac{\mathrm{Experimental}\;\mathrm{hydrogen}\;\mathrm{production}\;\mathrm{rate}}{\mathrm{Theoretical}\;\mathrm{hydrogen}\;\mathrm{production}\;\mathrm{rate}}$$

**Figure 5:**
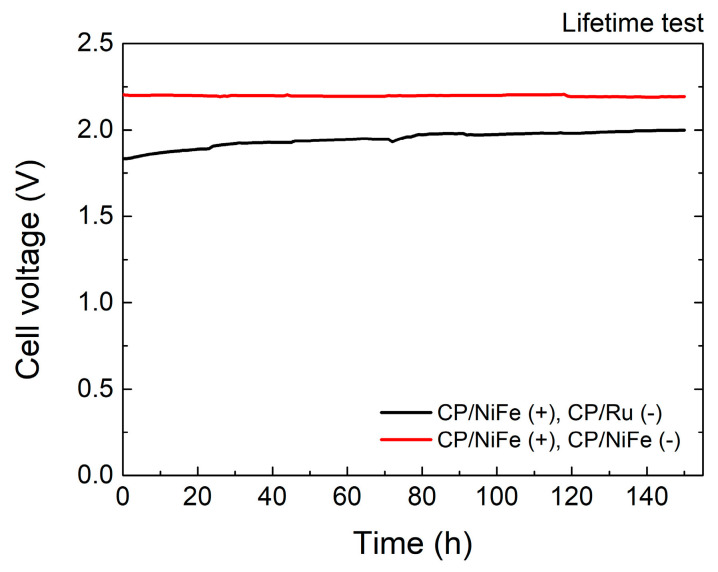
Stability of electrolyzer.

Figure 5 and Table 2 show the stability of the electrolyzer. In addition to having excellent catalytic performance, long-term stability is also a crucial factor to consider when manufacturing practical electrolyzers. The current density was fixed at 400 mA/cm^2^ to test the lifetime of the electrolyzer. After performing the test for 150 h, the cell voltage of NiFe(+)/Ru(−) increased by 0.167 V. This might be caused by the degradation of the catalysts. In comparison, NiFe(+)/NiFe(−) showed almost no decline after performing a test for 150 h. The cell voltage decreased slightly by 0.010 V.

### 3.2. Material Loading

Table 3 shows the material loading on CP [81]. This could be affected by the deposition of H_2_BDC-NH_2_ on the wall of the Teflon autoclave reactor. H_2_BDC-NH_2_ was used as the ligand for MOF. In this study, we used ethanol as an ecofriendly solvent instead of dimethyl formamide (DMF). CP is hydrophobic, with a water contact angle of 130.78°. Before the hydrothermal process, plasma treatment was used to make CP hydrophilic. This improves the contact between CP and the processing solutions. In this fashion, more material could be deposited on the CP.

### 3.3. SEM Inspection of Electrocatalysts

**Figure 6:**
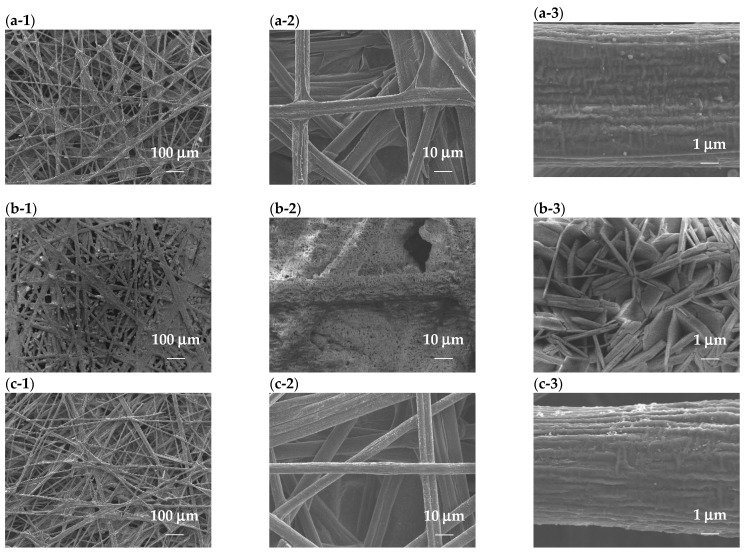
SEM images with 100×, 1000×, and 10,000× magnification. (**a-1**)–(**a-3**) CP, (**b-1**)–(**b-3**) CP/NiFe, and (**c-1**)–(**c-3**) CP/Ru.

Figure 6 shows SEM images of the CP-based electrocatalysts to investigate the surface morphology and distribution of metal ions. In the image of CP/NiFe, the SEM images show nanosheets grown on the CP. The nanoscale structure is a widely adopted strategy to enhance the performance of transition metal-based electrocatalysts [82,83]. The catalyst’s unique nano-pore array structure results in a larger active surface area and the ability to rapidly remove oxygen bubbles through the spatial confinement effect, both of which contribute to the catalytic effectiveness [84]. In the SEM images of grown Ru, many Ru particles still remain on the CP.

### 3.4. XRD Results of Electrocatalysts

**Figure 7:**
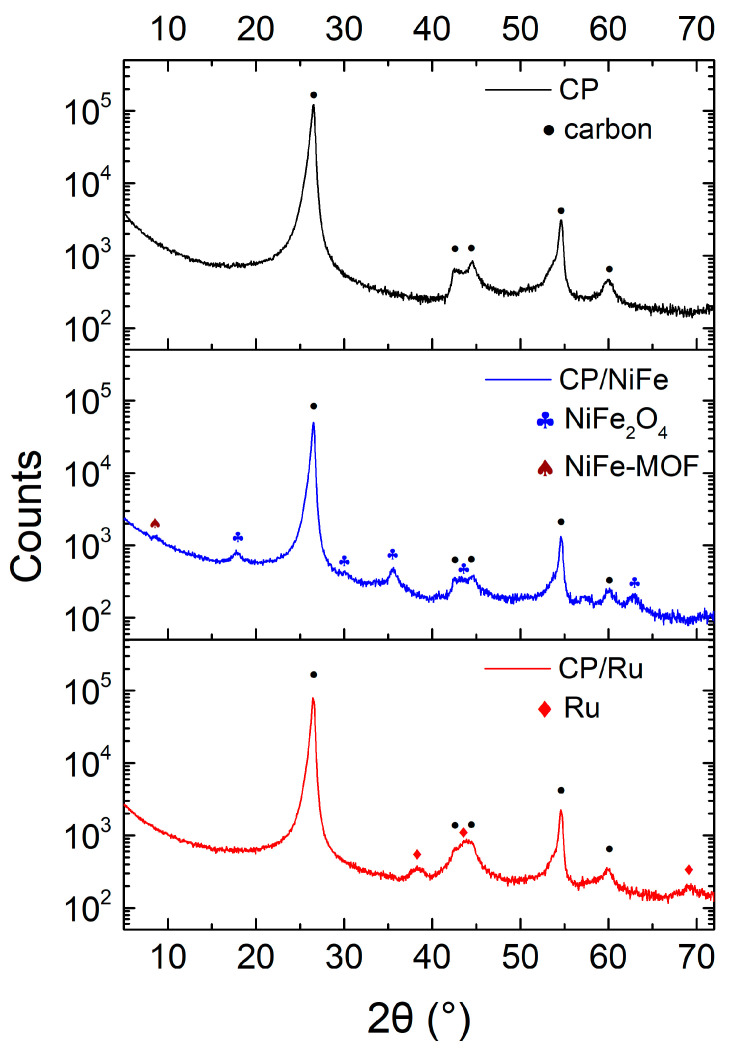
XRD spectrum of electrocatalysts.

XRD was used to analyze the crystal structure of the material. Figure 7 shows the XRD spectra of electrocatalysts. CP is mainly made of graphite, and it showed two primary peaks at 26.6° and 54.6° (JCPDS card no. 00-012-0212) [85]. Cui et al. showed that a new characteristic peak will appear at 8.8° when the NiFe-layered double hydroxide (NiFe-LDH) layer is completely turned into NiFe-MOF. A tiny peak at 8.8° corresponds to the (200) plane of NiFe-MOF [86]. CP/NiFe mainly exhibits a NiFe_2_O_4_ phase. The XRD spectra of CP/NiFe show five diffraction peaks at 17.8°, 30.1°, 35.6°, 58.1°, and 63.2° corresponding to the (111), (220), (311), (511), and (440) planes, respectively, of NiFe_2_O_4_ (JCPDS card no. 86-2667) [87]. In CP/Ru electrocatalysts, the XRD spectra show the hexagonal structure of Ru. The three diffraction peaks at 38.4°, 43.9°, and 69.5° corresponding to the (100), (101), and (110) planes, respectively, can be observed clearly (JCPDS card no. 89-3942) [88,89]. 

### 3.5. XPS Results of Electrocatalysts

**Figure 8:**
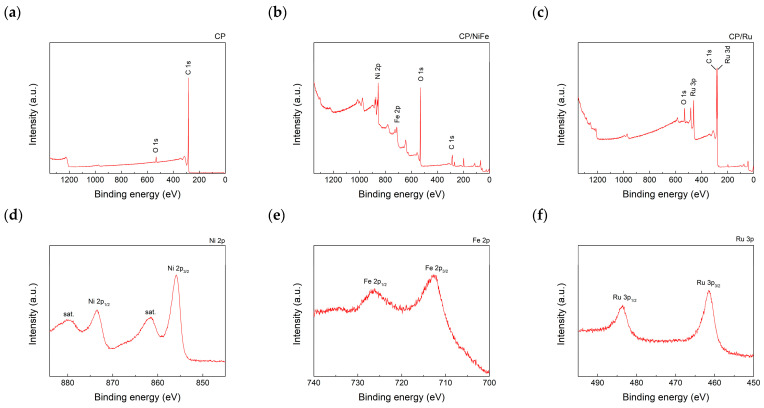
XPS wide-scan spectra of (**a**) CP, (**b**) CP/NiFe, and (**c**) CP/Ru. XPS fine-scan spectra of (**d**) Ni2p on CP/NiFe, (**e**) Fe2p on CP/NiFe, and (**f**) Ru3p on CP/Ru.

XPS was used to identify the elements on the CP surface. Figure 8a–c show the wide-scan spectrum, and Figure 8d–f show the fine-scan spectra of Ni, Fe on CP/NiFe, and Ru on CP/Ru. Ni2p_3/2_ and Ni2p_1/2_ peaks were seen at 855.9 eV and 873.5 eV, respectively [90,91]. Fe2p_3/2_ and Fe2p_1/2_ peaks were seen at 712.4 eV and 725.4 eV, respectively [91]. Ru3p_3/2_ and Ru3p_1/2_ peaks were seen at 461.9 eV and 484.3 eV, respectively [92]. The XPS results indicated that NiFe and Ru were successfully attached to the CP. Interestingly, although Ru was not clearly observed under SEM, the XPS results still show that Ru covers the CP. Ru has a significant intensity in Ru3p. The performance of the electrolyzer also strongly supported this observation. In the performance test, using CP/Ru as a cathode electrocatalyst resulted in a higher current density than using CP/NiFe, implying that Ru plays a significant role in the HER.

## 4. Conclusions

NiFe material and Ru were grown on CP as electrocatalysts in an alkaline water electrolysis system. For the NiFe catalyst, most of the phase is NiFe_2_O_4_, with a very small proportion of NiFe-MOF. In the NiFe(+)/Ru(−) electrolysis system, the current density reached 500 mA/cm^2^ at a cell voltage of 1.79 V, with a specific energy consumption of 4.9 kWh/m^3^ and an energy efficiency of 66.2%. In the NiFe(+)/NiFe(−) electrolysis system, the current density reached 500 mA/cm^2^ at a cell voltage of 2.23 V, with a specific energy consumption of 5.7 kWh/m^3^ and an energy efficiency of 56.6%. FE was 96–99% in both systems. The stability of NiFe(+)/NiFe(−) was high and did not decrease after running for 150 h under 400 mA/cm^2^. In this research, Ru material is used as the cathode catalyst to compare the performance differences between precious metals and non-precious metals. Although the performance of NiFe material is slightly poorer than Ru, the material cost in the laboratory is less than 10% of Ru. This indicated that NiFe_2_O_4_ material could be an option for low-cost, non-precious electrocatalysts for an alkaline water electrolysis system. This could be a key to effectively promoting large-scale applications of alkaline water electrolysis in the future.

## Figures and Tables

**Table 1:** Efficiency of electrolyzer.

Electrocatalysts	Current Density	Cell Voltage	Power Supply Voltage	H_2_ Production Rate (Theoretical)	H_2_ Production Rate (Experimental)	Specific Energy Consumption	Faradaic Efficiency (FE)	Energy Efficiency (η)
unit	mA/cm^2^	V	V	mL/min	mL/min	kWh/m^3^	%	%
NiFe(+)/Ru(−)	100	1.60	1.68	19.0	18.5	3.8	97.6	86.0
	500	1.79	2.21	94.9	93.8	4.9	98.8	66.2
	1000	1.98	2.84	189.8	181.8	6.5	95.8	49.9
NiFe(+)/NiFe(−)	100	2.02	2.08	19.0	18.5	4.7	97.5	69.4
	500	2.23	2.59	94.9	94.0	5.7	99.1	56.6
	1000	2.39	3.11	189.8	188.0	6.9	99.1	47.2

**Table 2:** Stability of electrolyzer.

Electrocatalysts	Time	Cell Voltage	Increment
unit	h	V	V
NiFe(+)/Ru(−)	0	1.833	
	50	1.936	0.103 (+5.6%)
	100	1.975	0.142 (+7.7%)
	150	2.000	0.167 (+9.1%)
NiFe(+)/NiFe(−)	0	2.203	
	50	2.197	−0.006 (−0.3%)
	100	2.201	−0.002 (−0.1%)
	150	2.193	−0.010 (−0.5%)

**Table 3:** Material loading on CP.

Electrocatalysts	Material Loading	Standard Deviation
CP/NiFe	2.03 mg/cm^2^	0.37 mg/cm^2^
CP/Ru	1.19 mg/cm^2^	0.14 mg/cm^2^

## Data Availability

Data are contained within the article.

## References

[1] Perera F., Ashrafi A., Kinney P., Mills D. (2019). Towards a fuller assessment of benefits to children’s health of reducing air pollution and mitigating climate change due to fossil fuel combustion. Environ. Res..

[2] Lu Y., Wu D., Qin Y., Xie Y., Ling Y., Ye H., Zhang Y. (2022). Facile construction of BiOBr/CoAl-LDH heterojunctions with suppressed Z-axis growth for efficient photoreduction of CO_2_. Sep. Purif. Technol..

[3] Tang W., Ye H., Xie Y., Chen P., Luo L., Zhang Y. (2023). Transition metal bismuth spheres dispersed and anchored in benzene-ring-grafted porous g-C_3_N_4_ nanosheets for photocatalytic reduction of CO_2_. Chem. Eng. J..

[4] Ahmed Z., Ahmad M., Murshed M., Shah M.I., Mahmood H., Abbas S. (2022). How do green energy technology investments, technological innovation, and trade globalization enhance green energy supply and stimulate environmental sustainability in the G7 countries?. Gondwana Res..

[5] Zaik K., Werle S. (2022). Solar and wind energy in Poland as power sources for electrolysis process-A review of studies and experimental methodology. Int. J. Hydrogen Energy.

[6] Gopinath M., Marimuthu R. (2022). A review on solar energy-based indirect water-splitting methods for hydrogen generation. Int. J. Hydrogen Energy.

[7] Mikovits C., Wetterlund E., Wehrle S., Baumgartner J., Schmidt J. (2021). Stronger together: Multi-annual variability of hydrogen production supported by wind power in Sweden. Appl. Energy.

[8] Gazey R., Salman S., Aklil-D’Halluin D. (2006). A field application experience of integrating hydrogen technology with wind power in a remote island location. J. Power Sources.

[9] Chaparro A., Soler J., Escudero M., De Ceballos E., Wittstadt U., Daza L. (2005). Data results and operational experience with a solar hydrogen system. J. Power Sources.

[10] Zou W.-J., Kim Y.-B. (2019). Temperature control for a 5 kW water-cooled PEM fuel cell system for a household application. IEEE Access.

[11] Noh C., Shin M., Kwon Y. (2022). A strategy for lowering cross-contamination of aqueous redox flow batteries using metal-ligand complexes as redox couple. J. Power Sources.

[12] Taherian R., Golikand A.N., Hadianfard M.J. (2012). Preparation and properties of a phenolic/graphite nanocomposite bipolar plate for proton exchange membrane fuel cell. ECS J. Solid State Sci. Technol..

[13] Ishaq H., Dincer I., Crawford C. (2022). A review on hydrogen production and utilization: Challenges and opportunities. Int. J. Hydrogen Energy.

[14] Chapman A., Itaoka K., Farabi-Asl H., Fujii Y., Nakahara M. (2020). Societal penetration of hydrogen into the future energy system: Impacts of policy, technology and carbon targets. Int. J. Hydrogen Energy.

[15] Pareek A., Dom R., Gupta J., Chandran J., Adepu V., Borse P.H. (2020). Insights into renewable hydrogen energy: Recent advances and prospects. Mater. Sci. Energy Technol..

[16] Tarhan C., Çil M.A. (2021). A study on hydrogen, the clean energy of the future: Hydrogen storage methods. J. Energy Storage.

[17] Li Y., Zhang T., Deng X., Liu B., Ma J., Yang F., Ouyang M. (2022). Active pressure and flow rate control of alkaline water electrolyzer based on wind power prediction and 100% energy utilization in off-grid wind-hydrogen coupling system. Appl. Energy.

[18] Arthur T., Millar G.J., Sauret E., Love J. (2023). Renewable hydrogen production using non-potable water: Thermal integration of membrane distillation and water electrolysis stack. Appl. Energy.

[19] Wu Z., Fang J., Liu N., Wu J., Kong L. (2021). The improvement in hydrogen storage performance of MgH_2_ enabled by multilayer Ti3C2. Micromachines.

[20] Chen Y.-S., Yang C.-W., Lee J.-Y. (2014). Implementation and evaluation for anode purging of a fuel cell based on nitrogen concentration. Appl. Energy.

[21] Jienkulsawad P., Patcharavorachot Y., Chen Y.-S., Arpornwichanop A. (2021). Energy and exergy analyses of a hybrid system containing solid oxide and molten carbonate fuel cells, a gas turbine, and a compressed air energy storage unit. Int. J. Hydrogen Energy.

[22] Millet P. (2015). Fundamentals of water electrolysis. Hydrog. Prod..

[23] Xiang R., Peng L., Wei Z. (2019). Tuning interfacial structures for better catalysis of water electrolysis. Chem.—Eur. J..

[24] Hu K., Fang J., Ai X., Huang D., Zhong Z., Yang X., Wang L. (2022). Comparative study of alkaline water electrolysis, proton exchange membrane water electrolysis and solid oxide electrolysis through multiphysics modeling. Appl. Energy.

[25] Theerthagiri J., Murthy A.P., Lee S.J., Karuppasamy K., Arumugam S.R., Yu Y., Hanafiah M.M., Kim H.-S., Mittal V., Choi M.Y. (2021). Recent progress on synthetic strategies and applications of transition metal phosphides in energy storage and conversion. Ceram. Int..

[26] Li J., Zhang B., Song Q., Xu X., Hou W. (2020). Sulfur and molybdenum Co-doped graphitic carbon nitride as a superior water dissociation electrocatalyst for alkaline hydrogen evolution reaction. Ceram. Int..

[27] Chi J., Yu H. (2018). Water electrolysis based on renewable energy for hydrogen production. Chin. J. Catal..

[28] Sun H., Yan Z., Liu F., Xu W., Cheng F., Chen J. (2020). Self-supported transition-metal-based electrocatalysts for hydrogen and oxygen evolution. Adv. Mater..

[29] Doan T.L., Lee H.E., Kim M., Cho W.C., Cho H.S., Kim T. (2022). Influence of IrO_2_/TiO_2_ coated titanium porous transport layer on the performance of PEM water electrolysis. J. Power Sources.

[30] Peng J., Chen Y., Wang K., Tang Z., Chen S. (2020). High-performance Ru-based electrocatalyst composed of Ru nanoparticles and Ru single atoms for hydrogen evolution reaction in alkaline solution. Int. J. Hydrogen Energy.

[31] Gao L., Cui X., Sewell C.D., Li J., Lin Z. (2021). Recent advances in activating surface reconstruction for the high-efficiency oxygen evolution reaction. Chem. Soc. Rev..

[32] Santos D., Šljukić B., Sequeira C., Macciò D., Saccone A., Figueiredo J. (2013). Electrocatalytic approach for the efficiency increase of electrolytic hydrogen production: Proof-of-concept using platinum–dysprosium alloys. Energy.

[33] Talluri B., Yoo K., Kim J. (2022). Lanthanum oxide rods as a novel and efficient bifunctional hydrogen and oxygen evolution electrocatalyst for overall water splitting. Ceram. Int..

[34] Naeem S., Naeem F., Mujtaba J., Shukla A.K., Mitra S., Huang G., Gulina L., Rudakovskaya P., Cui J., Tolstoy V. (2021). Oxygen generation using catalytic nano/micromotors. Micromachines.

[35] Levene J.I., Mann M.K., Margolis R.M., Milbrandt A. (2007). An analysis of hydrogen production from renewable electricity sources. Sol. Energy.

[36] Aulakh D.J.S., Boulama K.G., Pharoah J.G. (2021). On the reduction of electric energy consumption in electrolysis: A thermodynamic study. Int. J. Hydrogen Energy.

[37] Ensafi A.A., Jafari-Asl M., Nabiyan A., Rezaei B. (2016). Ni_3_S_2_/ball-milled silicon flour as a bi-functional electrocatalyst for hydrogen and oxygen evolution reactions. Energy.

[38] Mahale N.K., Ingle S.T. (2017). Electrocatalytic hydrogen evolution reaction on nano-nickel decorated graphene electrode. Energy.

[39] Liu Z., Corva M., Amin H.M., Blanc N., Linnemann J., Tschulik K. (2021). Single Co_3_O_4_ nanocubes electrocatalyzing the oxygen evolution reaction: Nano-impact insights into intrinsic activity and support effects. Int. J. Mol. Sci..

[40] Parkash A., Seehar T.H., Pirzada A.M., Islam M., Larik R. (2022). Evaluation of Novel Fuel Cell Catalysts with Ultra-Low Noble Metal Contents towards Electrochemical Catalysis. ECS J. Solid State Sci. Technol..

[41] Zhou D., Li P., Xu W., Jawaid S., Mohammed-Ibrahim J., Liu W., Kuang Y., Sun X. (2020). Recent advances in non-precious metal-based electrodes for alkaline water electrolysis. ChemNanoMat.

[42] Li Y., Deng X., Zhang T., Liu S., Song L., Yang F., Ouyang M., Shen X. (2023). Exploration of the configuration and operation rule of the multi-electrolyzers hybrid system of large-scale alkaline water hydrogen production system. Appl. Energy.

[43] Kim S., Han J.H., Yuk J., Kim S., Song Y., So S., Lee K.T., Kim T.-H. (2022). Highly selective porous separator with thin skin layer for alkaline water electrolysis. J. Power Sources.

[44] Gong M., Dai H. (2014). A mini review of NiFe-based materials as highly active oxygen evolution reaction electrocatalysts. Nano Res..

[45] Navadeepthy D., Rebekah A., Viswanthan C., Ponpandian N. (2021). Boosting the kinetics of oxygen and hydrogen evolution in alkaline water splitting using nickel ferrite/N-graphene nanocomposite as a bifunctional electrocatalyst. Int. J. Hydrogen Energy.

[46] Wang D., Watanabe F., Zhao W. (2017). Reduced graphene oxide-NiO/Ni nanomembranes as oxygen evolution reaction electrocatalysts. ECS J. Solid State Sci. Technol..

[47] Raja D.S., Lin H.-W., Lu S.-Y. (2019). Synergistically well-mixed MOFs grown on nickel foam as highly efficient durable bifunctional electrocatalysts for overall water splitting at high current densities. Nano Energy.

[48] Todoroki N., Wadayama T. (2019). Heterolayered Ni–Fe hydroxide/oxide nanostructures generated on a stainless-steel substrate for efficient alkaline water splitting. ACS Appl. Mater. Interfaces.

[49] Loh A., Li X., Taiwo O.O., Tariq F., Brandon N.P., Wang P., Xu K., Wang B. (2020). Development of Ni–Fe based ternary metal hydroxides as highly efficient oxygen evolution catalysts in AEM water electrolysis for hydrogen production. Int. J. Hydrogen Energy.

[50] Thangavel P., Kim G., Kim K.S. (2021). Electrochemical integration of amorphous NiFe (oxy) hydroxides on surface-activated carbon fibers for high-efficiency oxygen evolution in alkaline anion exchange membrane water electrolysis. J. Mater. Chem. A.

[51] Thangavel P., Ha M., Kumaraguru S., Meena A., Singh A.N., Harzandi A.M., Kim K.S. (2020). Graphene-nanoplatelets-supported NiFe-MOF: High-efficiency and ultra-stable oxygen electrodes for sustained alkaline anion exchange membrane water electrolysis. Energy Environ. Sci..

[52] Tseng C.-Y., Cheng I.-C., Chen J.-Z. (2022). Low-pressure-plasma-processed NiFe-MOFs/nickel foam as an efficient electrocatalyst for oxygen evolution reaction. Int. J. Hydrogen Energy.

[53] Liu C., Tseng C.-Y., Wang Y.-C., Cheng I.-C., Chen J.-Z. (2022). Low-Pressure Plasma-Processed Ruthenium/Nickel Foam Electrocatalysts for Hydrogen Evolution Reaction. Materials.

[54] Yang Y., Meng H., Yan S., Zhu H., Ma W., Wang C., Ma F., Hu Z. (2021). The in-situ construction of NiFe sulfide with nanoarray structure on nickel foam as efficient bifunctional electrocatalysts for overall water splitting. J. Alloys Compd..

[55] Ram Kumar K., Maiyalagan T. (2023). Iron nickel sulphide embedded on multi-walled carbon nanotubes as efficient electrocatalysts for oxygen evolution reaction in alkaline medium. Ceram. Int..

[56] Caprì A., Gatto I., Lo Vecchio C., Trocino S., Carbone A., Baglio V. (2023). Anion Exchange Membrane Water Electrolysis Based on Nickel Ferrite Catalysts. ChemElectroChem.

[57] Tetzlaff D., Pellumbi K., Baier D.M., Hoof L., Barkur H.S., Smialkowski M., Amin H.M., Grätz S., Siegmund D., Borchardt L. (2020). Sustainable and rapid preparation of nanosized Fe/Ni-pentlandite particles by mechanochemistry. Chem. Sci..

[58] Amin H.M., Apfel U.P. (2020). Metal-Rich Chalcogenides as Sustainable Electrocatalysts for Oxygen Evolution and Reduction: State of the Art and Future Perspectives. Eur. J. Inorg. Chem..

[59] Chen Z., Qing H., Zhou K., Sun D., Wu R. (2020). Metal-organic framework-derived nanocomposites for electrocatalytic hydrogen evolution reaction. Prog. Mater. Sci..

[60] Zhu Q.L., Xu Q. (2014). Metal-organic framework composites. Chem. Soc. Rev..

[61] Zhou H.C., Long J.R., Yaghi O.M. (2012). Introduction to metal-organic frameworks. Chem. Rev..

[62] Martinez-Lazaro A., Caprì A., Gatto I., Ledesma-García J., Rey-Raap N., Arenillas A., Espinosa-Lagunes F., Baglio V., Arriaga L. (2023). NiFe2O4 hierarchical nanoparticles as electrocatalyst for anion exchange membrane water electrolysis. J. Power Sources.

[63] Li T.-T., Shi B.-Y., Jiang L.-W., Zheng J.-F., Wang J.-J. (2022). Design and Preparation of NiFe_2_O_4_@ FeOOH Composite Electrocatalyst for Highly Efficient and Stable Oxygen Evolution Reaction. Molecules.

[64] Wang J., Cui W., Liu Q., Xing Z., Asiri A.M., Sun X. (2016). Recent progress in cobalt-based heterogeneous catalysts for electrochemical water splitting. Adv. Mater..

[65] Lu Z., Zhu W., Yu X., Zhang H., Li Y., Sun X., Wang X., Wang H., Wang J., Luo J. (2014). Ultrahigh hydrogen evolution performance of under-water “superaerophobic” MoS_2_ nanostructured electrodes. Adv. Mater..

[66] Zou X., Wu Y., Liu Y., Liu D., Li W., Gu L., Liu H., Wang P., Sun L., Zhang Y. (2018). In situ generation of bifunctional, efficient Fe-based catalysts from mackinawite iron sulfide for water splitting. Chem.

[67] Gao Y., Montana A., Chen F. (2017). Evaluation of porosity and thickness on effective diffusivity in gas diffusion layer. J. Power Sources.

[68] Waseem S., Maheshwari P.H., Maheshwari P., Sahu A.K., Saini A., Dhakate S.R. (2020). Configuring the Porosity and Microstructure of Carbon Paper Electrode Using Pore Formers and Its Influence on the Performance of PEMFC. Energy Fuels.

[69] Yao C., Zhang H., Liu T., Li X., Liu Z. (2012). Carbon paper coated with supported tungsten trioxide as novel electrode for all-vanadium flow battery. J. Power Sources.

[70] Lopez-Fernandez E., Sacedon C.G., Gil-Rostra J., Yubero F., Gonzalez-Elipe A.R., de Lucas-Consuegra A. (2021). Recent Advances in Alkaline Exchange Membrane Water Electrolysis and Electrode Manufacturing. Molecules.

[71] Razmjooei F., Morawietz T., Taghizadeh E., Hadjixenophontos E., Mues L., Gerle M., Wood B.D., Harms C., Gago A.S., Ansar S.A. (2021). Increasing the performance of an anion-exchange membrane electrolyzer operating in pure water with a nickel-based microporous layer. Joule.

[72] Carmo M., Fritz D.L., Mergel J., Stolten D. (2013). A comprehensive review on PEM water electrolysis. Int. J. Hydrogen Energy.

[73] Phillips R., Dunnill C.W. (2016). Zero gap alkaline electrolysis cell design for renewable energy storage as hydrogen gas. RSC Adv..

[74] Abbasi R., Setzler B.P., Lin S., Wang J., Zhao Y., Xu H., Pivovar B., Tian B., Chen X., Wu G. (2019). A roadmap to low-cost hydrogen with hydroxide exchange membrane electrolyzers. Adv. Mater..

[75] Du N., Roy C., Peach R., Turnbull M., Thiele S., Bock C. (2022). Anion-Exchange Membrane Water Electrolyzers. Chem. Rev..

[76] Vincent I., Bessarabov D. (2018). Low cost hydrogen production by anion exchange membrane electrolysis: A review. Renew. Sustain. Energy Rev..

[77] Reier T., Oezaslan M., Strasser P. (2012). Electrocatalytic oxygen evolution reaction (OER) on Ru, Ir, and Pt catalysts: A comparative study of nanoparticles and bulk materials. Acs Catal..

[78] Cherevko S., Geiger S., Kasian O., Kulyk N., Grote J.-P., Savan A., Shrestha B.R., Merzlikin S., Breitbach B., Ludwig A. (2016). Oxygen and hydrogen evolution reactions on Ru, RuO_2_, Ir, and IrO_2_ thin film electrodes in acidic and alkaline electrolytes: A comparative study on activity and stability. Catal. Today.

[79] Haynes W.M. (2014). CRC Handbook of Chemistry and Physics.

[80] Yu Z.Y., Duan Y., Feng X.Y., Yu X., Gao M.R., Yu S.H. (2021). Clean and affordable hydrogen fuel from alkaline water splitting: Past, recent progress, and future prospects. Adv. Mater..

[81] Zhu J., Hu L., Zhao P., Lee L.Y.S., Wong K.-Y. (2019). Recent advances in electrocatalytic hydrogen evolution using nanoparticles. Chem. Rev..

[82] Popczun E.J., McKone J.R., Read C.G., Biacchi A.J., Wiltrout A.M., Lewis N.S., Schaak R.E. (2013). Nanostructured nickel phosphide as an electrocatalyst for the hydrogen evolution reaction. J. Am. Chem. Soc..

[83] Wang Y., Zhang Y., Liu Z., Xie C., Feng S., Liu D., Shao M., Wang S. (2017). Layered double hydroxide nanosheets with multiple vacancies obtained by dry exfoliation as highly efficient oxygen evolution electrocatalysts. Angew. Chem. Int. Ed..

[84] Kim S., Ahn C., Cho Y., Hyun G., Jeon S., Park J.H. (2018). Suppressing buoyant force: New avenue for long-term durability of oxygen evolution catalysts. Nano Energy.

[85] Jiménez J.M.O., González R.L., Mendoza C.G., Cuauhtémoc-López I., Lemus M.A.A., Mendoza G.M. (2022). Comparative Effect of Adsorption and Photodegradation on Benzene and Naphthalene Using Bismuth Oxide Modify Graphene Oxide. J. Mex. Chem. Soc..

[86] Cui W., Bai H., Shang J., Wang F., Xu D., Ding J., Fan W., Shi W. (2020). Organic-inorganic hybrid-photoanode built from NiFe-MOF and TiO2 for efficient PEC water splitting. Electrochim. Acta.

[87] Zhang J., Chen Z.-H. (2020). Preparation and electrochemical properties of mesoporous NiFe_2_O_4_/N-doped carbon nanocomposite as an anode for lithium ion battery. Front. Mater..

[88] Gopinath K., Karthika V., Gowri S., Senthilkumar V., Kumaresan S., Arumugam A. (2014). Antibacterial activity of ruthenium nanoparticles synthesized using *Gloriosa superba* L. leaf extract. J. Nanostruct. Chem..

[89] Kotsugi Y., Han S.-M., Kim Y.-H., Cheon T., Nandi D.K., Ramesh R., Yu N.-K., Son K., Tsugawa T., Ohtake S. (2021). Atomic layer deposition of Ru for replacing Cu-interconnects. Chem. Mater..

[90] Cai M., Cao S., Zhuo Z., Wang X., Shi K., Cheng Q., Xue Z., Du X., Shen C., Liu X. (2022). Fabrication of Ni_2_P Cocatalyzed CdS Nanorods with a Well-Defined Heterointerface for Enhanced Photocatalytic H_2_ Evolution. Catalysts.

[91] Zhang F.-S., Wang J.-W., Luo J., Liu R.-R., Zhang Z.-M., He C.-T., Lu T.-B. (2018). Extraction of nickel from NiFe-LDH into Ni 2 P@ NiFe hydroxide as a bifunctional electrocatalyst for efficient overall water splitting. Chem. Sci..

[92] Morgan D.J. (2015). Resolving ruthenium: XPS studies of common ruthenium materials. Surf. Interface Anal..

